# A Comparison of McGrath Mac and HugeMed Video Laryngoscopes in Pediatric Patients Under 3 Years Old—A Prospective Randomized Trial

**DOI:** 10.3390/healthcare13070842

**Published:** 2025-04-07

**Authors:** Gamze Tanirgan Cabakli, Kemal Tolga Saracoglu, Ruslan Abdullayev, Ecem Guclu, Pawel Ratajczyk, Ayten Saracoglu

**Affiliations:** 1 Department of Anesthesiology and Reanimation, Marmara University Medical School, 34899 Istanbul, Turkey; gamze.cabakli@istanbul.edu.tr (G.T.C.); ruslan.abdullayev@marmara.edu.tr (R.A.); ecem.ozturk@marmara.edu.tr (E.G.); 2 Department of Anesthesiology, College of Medicine, University of Florida, UF Health, Jacksonville, FL 32209, USAsaracogluayten@gmail.com (A.S.); 3 Department of Anesthesiology and Intensive Therapy, Medical University of Lodz, 90-153 Lodz, Poland

**Keywords:** pediatric anesthesia, videolaryngoscopes, endotracheal intubation, laryngoscopy, complications

## Abstract

Background: Children generally face a higher incidence of airway management complications, intubation difficulties, and the risk of failed intubation. Currently, there is sufficient evidence in clinical practice for the use of videolaryngoscopes in pediatric airway management. However, there are a number of standard-blade videolaryngoscopes available for children. In addition, there is no clear recommendation on which videolaryngoscope is superior. The primary objective of this study is to compare the first pass success rate and the Percentage of Glottic Opening (POGO) scores with Cormack–Lehane (CML) scores obtained through direct and indirect laryngoscopy with HugeMed and McGrath Mac videolaryngoscopes in pediatric patients with an unanticipated, difficult airway. Materials and Methods: Following the Ethics Committee approval and written parental consents, a total of 40 elective surgical patients, aged 3 and under, with ASA 1–3 risk classification, and undergoing general anesthesia, were included in the study. After induction of general anesthesia, the first group of patients (Group McGrath, *n* = 20) was intubated with the McGrath Mac videolaryngoscope, and the second group (Group HugeMed, *n* = 20) with the HugeMed videolaryngoscope. Before intubation, CML and POGO scores were recorded for both groups using direct and indirect laryngoscopy with videolaryngoscopes. Intubation time, number of attempts, need for cricoid pressure, optimization maneuver requirement, and hemodynamic parameters were recorded for both groups. Results: There was no significant difference between groups in demographic data including age, gender, body mass index, ASA, and hemodynamic parameters. A significant improvement was observed in CML and POGO scores using indirect laryngoscopy (*p* < 0.001). CML scores obtained with the McGrath Mac were significantly lower than the HugeMed Group (*p* = 0.0034). The mean POGO value calculated with indirect laryngoscopy was significantly higher in the McGrath Group compared to the HugeMed Group (92.63 ± 6.09 vs. 88.75 ± 4.44, respectively). Conclusions: Videolaryngoscopes improved laryngeal visualization in children under 3 years old. Compared to HugeMed, in indirect laryngoscopy, the McGrath Mac videolaryngoscope was found to be superior, with better CML and POGO scores. However, number of tracheal intubation attempts, success rate, complication risk, and hemodynamic parameters did not show any significant difference between the groups. Clinical trial registration number was NCT06484517.

## 1. Introduction

Children are generally at a higher risk of airway management complications, intubation difficulties, and the risk of failed intubation [1]. These risks are associated with increased morbidity and mortality rates, increased frequency of intensive care admissions, and increased cardiovascular instability in children with difficult airway [2]. Currently, there is sufficient evidence to suggest the use of videolaryngoscopes (VLs) in pediatric airway management, and they are increasingly used in clinical practice [3]. The French guidelines for the management of a child’s airway under anesthesia recommend the use of a videolaryngoscopy as the first option in cases of anticipated difficult intubation [4]. The recently published joint guidelines on airway management in neonates and infants also recommended VLs as the first stage for both the clinically stable and unstable patients [5]. In particular, the COVID-19 pandemic has had a significant impact on VLs. Both the availability of videolaryngoscopes and their routine use in all cases have significantly increased [6]. It has been reported that standard blades are superior to non-standard blades in pediatric patients [7]. However, there are many standard bladed VLs available in the market for children. Therefore, a clear recommendation cannot be made regarding which VL is superior [8]. Many prospective comparative studies have been conducted in an attempt to find the “best” VL, but often conflicting results have been obtained [9]. McGrath Mac is a VL with a standard blade and a high-resolution camera at the tip [10]. A disposable plastic blade and a battery-contained handle are included [11]. As it has an external display monitor, there is no need for additional cable, screen, or power unit connections [12].

The HugeMed VL features a reusable Macintosh blade. It has a 3.5-inch HD screen at the top of the handle. The screen can rotate right-left and up-down. The camera at the tip of the blade has a field of view of 60 degrees [6]. Despite the frequent use of both McGrath Mac and HugeMed VL in pediatric patients in recent years, there is currently no prospective comparative study in the literature comparing the two VLs. The primary objective of this study was to compare first pass success rate and POGO scores reflecting the percentage of glottis opening in unexpected difficult pediatric airways using HugeMed and McGrath Mac VLs for both direct and indirect laryngoscopy. We aimed to compare Cormack Lehane scores as well. Our secondary objectives included the postoperative complication rate and their impact on hemodynamic response.

## 2. Materials and Methods

**Figure 1:**
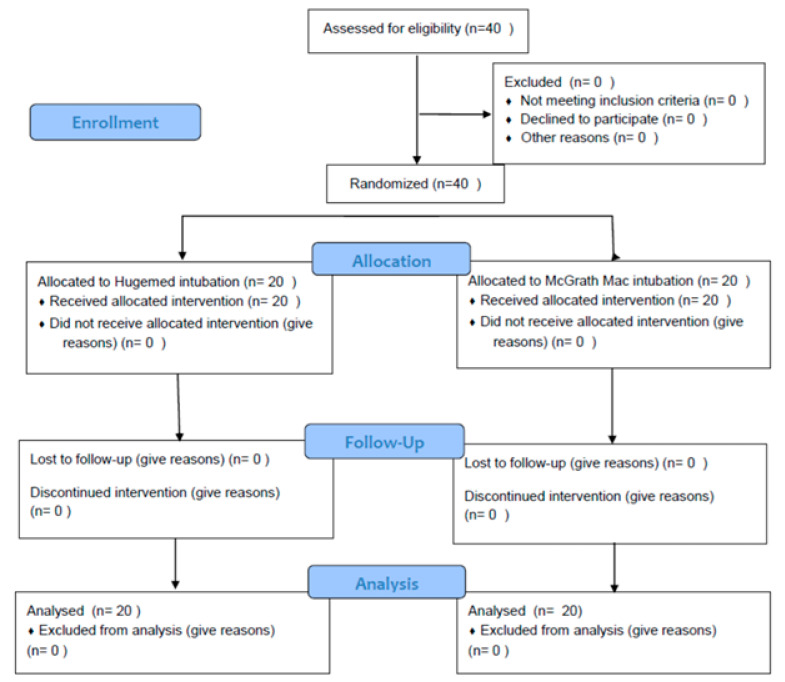
Flow diagram.

This is a randomized controlled trial, and Ethics Committee approval from the Institutional Review Board of our university hospital (09.2021.961) was obtained. The study was retrospectively registered on www.clinicaltrials.gov (Registration No: NCT06484517). A total of 40 elective surgical patients under the age of 3, classified as American Society of Anesthesiologists (ASA) 1–3 risk group, who underwent general anesthesia with predicted normal airways were included in the study. Patients were divided into two groups using a sealed envelope method as McGrath Group and HugeMed Group. The flow diagram is presented in Figure 1. After standard anesthesia monitoring to each group, general anesthesia was administered. Written informed consent was obtained from the parents of the patients.

### 2.1. Exclusion Criteria

Patients who could not obtain parental consent, those classified as ASA 4 and above, individuals with serious cardiac and respiratory problems, patients undergoing laryngeal and oral surgery and anticipated to have a difficult airway were not included in the study.

### 2.2. General Anesthesia

Thirty minutes before anesthesia induction, all patients received premedication with intravenous 0.03 mg/kg midazolam. Standard monitoring before surgery included continuous ST-segment analysis with a 3-lead electrocardiogram (ECG), peripheral oxygen saturation (SpO_2_), and intermittent non-invasive blood pressure assessment. After three minutes of preoxygenation with 100% oxygen, general anesthesia was induced with intravenous propofol 3 mg/kg following the administration of fentanyl 2 µg/kg. Muscle relaxation was achieved in all patients with intravenous rocuronium 0.5 mg/kg.

### 2.3. Tracheal Intubation

**Figure 2:**
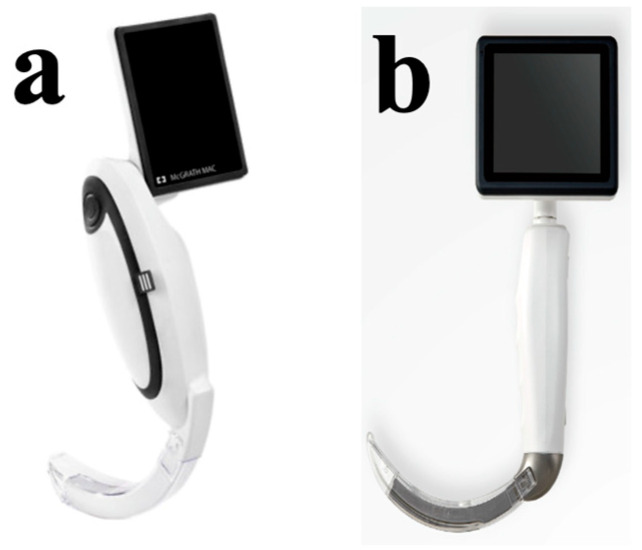
MacGrath Mac (**a**) and HugeMed videolaryngoscope (**b**).

A direct laryngoscopy provides a clear visualization of the larynx under direct vision. An indirect laryngoscopy uses a camera to visualize the larynx without a direct view. Both McGrath Mac and HugeMed laryngoscopes are devices with an attached camera system (Figure 2). When the camera is off, the device functions as a direct laryngoscope. When turned on, it operates as an indirect laryngoscope. In this way, they integrate the advantages of both direct laryngoscopy and indirect video laryngoscopy.

After general anesthesia induction, Group McGrath patients were intubated with the McGrath Mac VL (Medtronic, Minneapolis, MN, USA). Before intubation, CML and POGO scores were recorded by applying direct and indirect laryngoscopy. All tracheal intubations were performed by the same expert with over 10 years of experience in pediatric anesthesia. Rocuronium was administered at a dose of 0.6 mg/kg as a muscle relaxant for tracheal intubation during anesthesia induction. The train-of-four measurement was conducted every 10 s at a train frequency of 0.1 Hz after administering rocuronium. Laryngoscopy was performed once the count reached zero.

For the second group (Group HugeMed, *n* = 20), the HugeMed VL (Shenzhen HugeMed Medical Technical Development Co., Ltd., Shenzhen, China) was used. After the induction of general anesthesia, patients underwent direct and indirect laryngoscopy with HugeMed VL under the guidance of train-of-four monitoring. Patients were intubated using one of the appropriate Macintosh blades numbered 1, or 2, based on their height, weight, and age in both the McGrath and HugeMed groups. Hyperangulated blades were not used. The number of attempts, the need for cricoid pressure, optimization maneuvers, success rate, and hemodynamic parameters of both groups were recorded. The optimization maneuvers were used to enhance visualization of the glottis and improve intubation success rate during videolaryngoscopy. These maneuvers included blade tip manipulation for direct epiglottic lifting, withdrawal maneuver to pull back the blade, BURP Maneuver (Backward, Upward, Rightward Pressure) as an external laryngeal manipulation and rotating the tracheal tube 90° counterclockwise. Both VLs were initially used as direct laryngoscopes; the camera system was not activated, and CML and POGO scoring were performed. A second anesthetist other than the operator, took a photograph of the laryngeal view. Following the photo capture during direct laryngoscopy, patients were ventilated with a face mask and 100% oxygen for 1 min. Subsequently, indirect laryngoscopy was performed, and another photograph was taken from the image on the VL integrated camera. An iPhone X brand mobile phone camera (Apple Inc., Cupertino, CA, USA) was used for photographing. Another researcher, blinded to the type of VL used, conducted the CML and POGO scoring based on the photos. After the second photo shoot, children were intubated with an appropriately sized tracheal tube. Optimization maneuvers, including cricoid pressure and cricoid manipulation, were recorded to determine whether we had performed them.

### 2.4. POGO Classification

The POGO classification was based on a 100% scale, where a score of 100% indicated a full glottic opening from the anterior commissure to the posterior cartilage. The absence of a glottic opening was scored as 0.

Patient characteristics, including age, gender, body weight, Mallampati score, mouth opening, thyromental distance, ASA physical status classification, and intubation time, were recorded. Intubation time was defined as the time from the holding time of laryngoscope to the visualization of end-tidal carbon dioxide (EtCO_2_). Additionally, mean arterial pressure, heart rate, SpO_2_, and EtCO_2_ were recorded before anesthesia induction, after induction, and post-intubation. The number of intubation attempts and complications were also recorded for both groups including throat soreness, bronchospasm, laryngospasm, mucosal trauma, dental injury, arrhythmia, hemodynamic changes, pulmonary aspiration, and mortality.

### 2.5. Statistical Analysis

Data were analyzed using IBM SPSS V23. Normality was assessed using the Shapiro–Wilk test. Independent samples *t*-test was used for normally distributed data when comparing between two groups, and the Mann–Whitney U test was used for non-normally distributed data. Repeated measures analysis of variance (ANOVA) was used for normally distributed data within groups over time, with multiple comparisons assessed using the Bonferroni test. Friedman test was used for non-normally distributed quantitative data within groups over time, and multiple comparisons were assessed using the Dunn test. Wilcoxon test was used to compare non-normally distributed POGO values within groups for VL and DL. The Marginal Homogeneity Test was used to compare VL and DL Cormack–Lehane scores within groups. Chi-square test and Yates correction were used to compare categorical data between groups. Relationships between non-normally distributed variables were examined using Spearman’s rho correlation coefficient. The significance level was set at *p* < 0.050.

### 2.6. Sample Size Calculation

The sample size was calculated based on data from a similar comparative study [13]. As the primary outcomes were the comparison of first attempt success rate, CML, and POGO scores, a two-tailed effect size of 1.175 was calculated with alpha (α) set at 0.05 and beta (β) at 0.10 when we considered a significant decrease between the two groups. Accordingly, it was determined that 19 patients should be included in each group. Taking into account a potential 20% loss of patient data, we planned to include 20 patients in each group, totaling 40 patients.

## 3. Results

The mean age was 28.47 ± 7.83 months for McGrath Group and 23.43 ± 7.38 months for HugeMed Group (*p* = 0.054). In one patient, a postoperative severe airway edema, following an unexpectedly prolonged surgery, led to cardiac arrest after extubating. The child had a large cleft palate and did not respond to cardiopulmonary resuscitation and, unfortunately, did not survive. As a result, this case was excluded from the study. Data from a total of 39 patients were analyzed, and it is noteworthy that all patients were successfully intubated on the first attempt.

Ten of our patients were admitted to the orthopedic ward and underwent surgery for orthopedic reasons. Three of them had humerus fractures, one had debridement, four had tendon repairs, and two had plate removal surgeries. Nine patients were operated on by the neurosurgery team. Two of them had posterior fossa surgeries, four had extraventricular drain placements, and three had shunt surgeries. Six patients were operated on by the pediatric surgery team. One of them had stoma closure, two had circumcision, two had appendectomies, and one had a cystoscopy. Two patients underwent cleft palate surgery by the plastic surgery team. Thirteen patients underwent surgery by the ear, nose, and throat (ENT) team. Six of them had tonsillectomies, five had cochlear implants, and two had cochlear implant revisions. When comparing the demographic data, including age, gender, body mass index, and ASA classification, no significant differences were observed between the groups (*p* > 0.05, Table 1).

As postoperative complications, sore throat was observed in 1 patient in the HugeMed Group and bronchospasm was observed in 1 patient in the McGrath group. Laryngospasm and minimal mucosal bleeding were noted in one patient in each group (Table 1). Minimal mucosal bleeding was observed in patients with a higher frequency of intubation attempts. These were identified upon seeing blood on the laryngoscope blade. Upon oral inspection in these patients, no actively bleeding focus was observed. After oral aspiration, pressure was applied with a sponge to the areas where minimal bleeding was observed for 1 min. Following extubating, the oral cavity was inspected again with the aid of light, and it was confirmed that there was no bleeding.

After the induction of anesthesia, the number of tracheal intubation attempts were similar in both groups (*p* = 0.788, Table 2). The mean tracheal intubation time for Group McGrath was 39.26 ± 21.88 s, while those for Group HugeMed were 48.07 ± 27.15 s. No significant difference was observed between the two groups (*p* > 0.05). The optimization maneuvers were performed in six patients from Group McGrath (30%). The maneuvers included tracheal tube rotation in three patients, lifting the epiglottis instead of tongue base in two patients, and a withdrawal maneuver in one patient. The HugeMed Group experienced optimization maneuvers in five patients (25%). There was no statistically significant difference between groups (*p* > 0.05). One patient required a BURP maneuver, while two patients required tracheal tube rotation. Epiglottis lifting was performed for two patients.

The comparison of successful tracheal intubation attempts did not show any significant difference between groups (χ^2^ = 0.022, *p* = 0.882, Table 3).

The Modified Cormack–Lehane scores obtained from direct laryngoscopy before intubation were similar between the two groups (*p* = 0.462, Table 4). When comparing the CML scores from direct laryngoscopy with indirect laryngoscopy values, both groups showed a significant improvement in CML scores with indirect laryngoscopy (*p* < 0.001). The CML scores obtained with McGrath Mac VL were significantly lower than those in the HugeMed Group (*p* = 0.0034, Table 4). The mean difference was −0.5 (95% CI: −0.8 to −0.2), with a Cohen’s d of −0.65, indicating a medium effect size.

The mean POGO values measured during direct laryngoscopy were similar between the McGrath and HugeMed groups (78.42 ± 12.48 and 80.75 ± 6.74, respectively). The mean POGO values calculated with indirect laryngoscopy using both VLs were significantly higher than those obtained from direct laryngoscopy (*p* < 0.001, Table 4). The mean POGO value calculated with indirect laryngoscopy in the McGrath Group was significantly higher than that in the HugeMed Group (92.63 ± 6.09 vs. 88.75 ± 4.44, respectively). The mean difference was 3.88 (95% CI: 1.12 to 6.64), with a Cohen’s d of 0.75, indicating a medium to large effect size. The success rate for the first intubation attempt was 52.6% in the McGrath group and 55.0% in the HugeMed group. The odds ratio for success in the McGrath group compared to the HugeMed group was 0.91 (95% CI: 0.45 to 1.85), indicating no significant difference.

**Figure 3:**
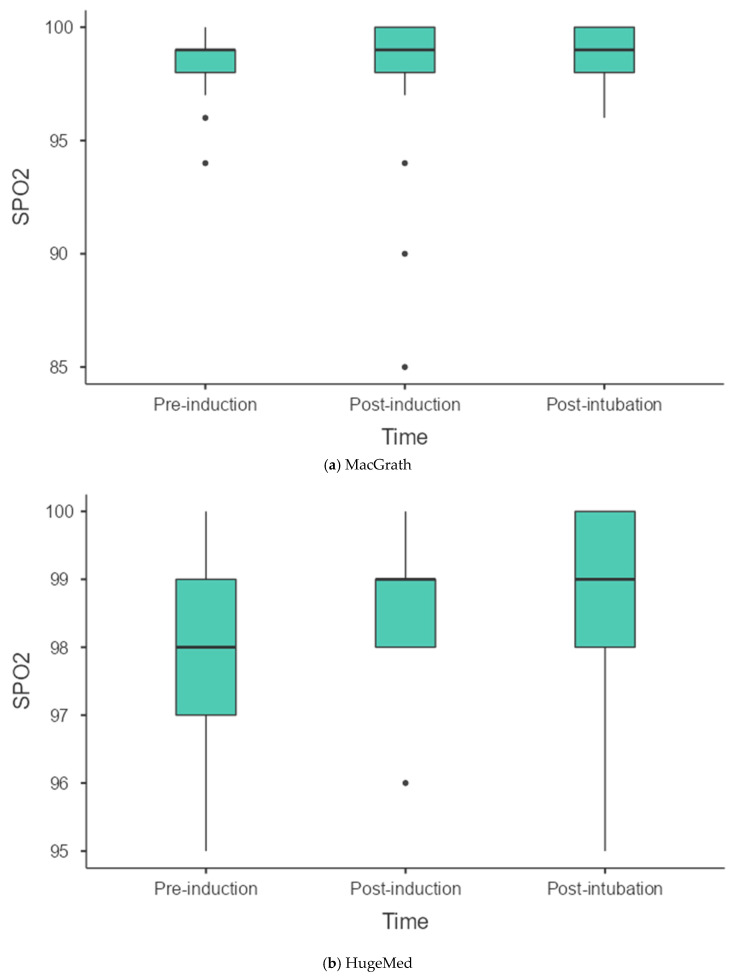
Box plot graphs of the SpO_2_ change over time in the groups. Dots represent the outliers. (**a**) MacGrath. (**b**) HugeMed.

There was no significant difference between and within groups in systolic, diastolic and mean arterial pressure, and heart rate immediately before induction, within two minutes after induction, and post-intubation period until the patient was discharged from the operating room (*p* > 0.05). There was no significant difference in the mean oxygen saturation values before induction, after induction, and post-intubation between the groups (*p* > 0.05). However, in the HugeMed Group, there was a significant difference in oxygen saturation values before induction and post-intubation (*p* = 0.014). In Group McGrath, no significant difference was observed in oxygen saturation values over time (*p* = 0.420). There was no difference in mean end-tidal carbon dioxide values before and after induction between the groups (*p* > 0.05). However, there was a significant difference in post-intubation end-tidal carbon dioxide values between the groups (*p* = 0.039). In Group HugeMed, the mean end-tidal carbon dioxide was 32.10 mmHg, while in Group McGrath, it was 35.74 mmHg. Additionally, in both Group HugeMed and Group McGrath, there was a significant difference between pre-induction end-tidal carbon dioxide values and post-intubation end-tidal carbon dioxide values (*p* < 0.001, Table 5). The box plot graphs of the SpO_2_ change over time in the groups are shown in Figure 3.

Within each group, no statistically significant relationship was found between the number of attempts and post-intubation systolic, diastolic, mean arterial pressure, heart rate, oxygen saturation, and end-tidal carbon dioxide values (*p* > 0.05, Table 6).

An insignificant positive relationship was observed between age and POGO scores with a VL and a negative relationship between POGO scores with a direct laryngoscopy in the McGrath Group. It was observed that, as the age increased, the POGO score with a direct laryngoscope decreased, while the POGO score with VL increased. In the HugeMed group, a positive insignificant relationship was observed between the age and POGO scores with VL and POGO with direct laryngoscopy. We observed that POGO scores with VL and direct laryngoscopy decreased as age increased (Table 7).

## 4. Discussion

In this prospective randomized controlled study, data obtained through direct and indirect laryngoscopy with HugeMed and McGrath VLs were compared in children under 3 years of age undergoing elective surgery requiring tracheal intubation. Significant improvements in both CML and POGO scores were recorded with indirect laryngoscopy in both groups compared to direct laryngoscopy. The McGrath VL yielded higher POGO scores and lower CML values compared to HugeMed, however the number of intubation attempts, success and complication rates, or hemodynamic data did not differ.

In recent years, videolaryngoscopy has increasingly been regarded as a primary tool for airway management, rather than merely a backup for difficult intubations. However, it does not guarantee a 100% success rate in intubation [14]. Although videolaryngoscopy offers numerous benefits, there are potential challenges that can arise, many of which can be easily mitigated with proper knowledge and attention. Adequate training, consistent practice, and familiarity with device-specific tools are essential to ensure that the enhanced laryngeal view leads to successful tracheal intubation. Our study demonstrated that videolaryngoscopy improves laryngeal visualization, with the McGrath VL showing a particularly enhanced image. However, the clarity of vocal cord visibility did not influence either the rate of tracheal intubation attempts or the success of the first attempt. Various factors contribute to the number of intubation attempts, with the most critical being the ability to direct the tracheal tube. In our study, the anesthesiologists were experienced with both devices, which used a standard Macintosh-type blade. Despite the team’s expertise and group standardization, first-pass failure occurred in nine patients using both VLs. We believe that further practice and experience in this area will help reduce the number of intubation attempts.

The results from the PeDI Registry suggested that we still do not make accurate choices for pediatric airway management [15]. In the analysis of difficult airway cases in children, 46% of anesthetists chose direct laryngoscopy as the first-choice airway technique, with a success rate of 3%. Only 18% of anesthesia experts chose videolaryngoscopy, reporting a success rate of 55%. While the physiological changes in children’s airways and the associated risk of intubation difficulty are known, the superiority of VLs over direct laryngoscopy has been proven in many studies [16]. However, recent multicenter survey studies have revealed that 88% of direct laryngoscopes are routinely used in the intubation of children, with only 11% preferring VLs for all intubations [17].

One advantage of videolaryngoscopes is the ability to obtain both direct and indirect images. A meta-analysis from 2014 comparing pediatric videolaryngoscopes with direct laryngoscopes in 14 randomized controlled studies reported better visualization of the glottis with VLs [18]. Although the initial success rate and the associated complications were similar for both laryngoscope types, this meta-analysis reported a higher incidence of failure for VLs. It is evident that more experience with pediatric VLs has been gained over the years. A Cochrane meta-analysis in 2017, including 803 children, reported marked heterogeneity among studies on this topic and emphasized the difficulty in outcome analysis [19]. As reported in the Anaesthesia Practice In Children Observational Trial (APRICOT), prospective multicenter observational study, education, training, and research can improve perioperative care and reduce complication rates in these children [20]. Considering these factors, in this study, an anesthetist with over 10 years of pediatric anesthesia experience was dedicated to tracheal intubation. The results showed that not only were laryngeal structures better visualized with VLs, but the success rate of tracheal intubation was also 100% in experienced hands. Additionally, we observed only minor, manageable complications through this approach.

Our study demonstrated a significant improvement in both CML and POGO scores with indirect laryngoscopy. Standard-blade videolaryngoscopes resemble traditional direct laryngoscopes in shape and design, requiring less experience and being easy to learn [7]. However, non-standard blades should be used by experienced operators. Despite the recommendation for the use of standard-blade VLs, there is no clear suggestion in the literature regarding which standard-blade VLs can be used in pediatric patients.

Even though the operators in our study were experienced in pediatric anesthesia, we observed minor complications including sore throat, bronchospasm, and minimal mucosal bleeding. Postoperative sore throat was observed in one patient from the HugeMed Group. It was likely due to mechanical irritation from the laryngoscope blade. Despite using the same blade sizes, slight variations between laryngoscopes may have contributed to this outcome. Typically mild and self-limiting, it can still impact patient comfort and recovery. Therefore, operators should consider this possibility during tracheal intubation. Minimal mucosal injuries might occur due to minor trauma from laryngoscope insertion, particularly in difficult airways. Typically they are minor and self-resolving but could indicate excessive force or a suboptimal technique.

McGrath Mac VL, on the market since 2010, has had conflicting results in studies conducted in children. In a study involving 30 children aged 1–10 years undergoing surgical release of torticollis, McGrath Mac was compared with Macintosh direct laryngoscope [10]. The McGrath group reported a significantly prolonged intubation time: 31.4 ± 6.7 s vs. 26.1 ± 5.4 s. In another pediatric manikin study comparing McGrath Mac to direct laryngoscopy, the use of McGrath Mac VL increased intubation success in cases with a high probability of difficult intubation [1]. In another manikin study by Owada et al. [13] in 2017, the authors revealed that intubation with Airtraq was more successful than with McGrath Mac and Macintosh laryngoscopes. Intubations with Airtraq had lower CML scores, higher POGO scores, and less postoperative throat pain and dental trauma compared to intubations with the other two laryngoscopes.

In our study, tracheal intubation attempts were performed lifting the tongue base. Kucukoglu et al. [21] compared McGrath and Miller laryngoscopes when the direct lifting method of the epiglottis was used. Both laryngoscopes demonstrated comparable effectiveness in terms of POGO and CL scores. In a randomized controlled study, the McGrath Mac was compared with a direct Macintosh laryngoscope. Similarly, there were again no significant differences in the POGO scores between the two groups [22]. A unique aspect of our study was the comparison of both direct and indirect visualization methods. This approach allowed us to gain a clearer understanding of which method may be more beneficial for the pediatric population.

HugeMed is a recently introduced VL that offers adult and pediatric blade sizes, and its use has increased with the COVID-19 pandemic. To the best of our knowledge, this study is the first to investigate pediatric HugeMed VL. We chose McGrath Mac VL as the comparison group because it is easily accessible and we have experience with it. While the success rate and complication rate did not differ, the results of our study clearly demonstrated the superiority of McGrath Mac VL over HugeMed in visualizing pediatric laryngeal structures.

## 5. Limitations

Limitations of this study include its single-center nature and the low sample size. Despite the low sample size, meaningful results that can shed light on the use of VLs in our clinical practice have been obtained. However, it is clear that there is a need for further comparative multicenter studies. We performed a second exposure of glottis and the photos were recorded. This approach could increase the risk of a laryngeal edema and extended intubation time, which can lead to a lower peripheral oxygen saturation and decrease the patients benefit. However, no complication occurred. There was a time period of 1 min between the direct and indirect laryngoscopies. This could affect the POGO score and CML grade. Finally, we did not measure the ease of the tracheal tube passage. This can be a study parameter for the planned future studies.

## 6. Conclusions

In conclusion, VLs improved laryngeal visualization in elective surgical pediatric patients under 3 years old. McGrath Mac VL, compared to HugeMed in indirect laryngoscopy, was found to be superior with better CML and POGO scores. However, the number of tracheal intubation attempts, success rate, complication risk, and hemodynamic parameters did not show significant differences.

## Figures and Tables

**Table 1:** Comparison of demographic data and complications between groups.

	Group		Total	Test Syt.	*p*
	HugeMed	McGrath			
Gender					
Male	10 (50.00)	9 (47.37)	19 (48.72)	0.000	1.000 **
Female	10 (50.00)	10 (52.63)	20 (51.28)		
ASA					
1	3 (15.00)	12 (63.16)	15 (38.46)	10.048	0.007 *
2	12 (60.00)	6 (31.58)	18 (46.15)		
3	5 (25.00)	1 (5.26)	6 (15.38)		
Mallampati Score					
1	5 (25.00)	5 (26.32)	10 (25.64)	0.685	0.710 *
2	11 (55.00)	12 (63.16)	23 (58.97)		
3	4 (20.00)	2 (10.53)	6 (15.38)		
Sore throat					
Yes	1 (5.00)	0 (0.00)	1 (2.56)	---	---
No	19 (95.00)	19 (100.00)	38 (97.44)		
Complication					
Bronchospasm	0 (0.00)	1 (5.26)	1 (2.56)	1.093	0.779 *
Laryngospasm	1 (5.00)	1 (5.26)	2 (5.13)		
Minimal bleeding	1 (5.00)	1 (5.26)	2 (5.13)		
None	18 (90.00)	16 (84.21)	34 (87.18)		

* Chi-square test, ** Yates correction, frequency. ASA: American Society of Anesthesiologists.

**Table 2:** The comparison of the number of tracheal intubation attempts.

Descriptives				
		Percentiles		
	Group	25th	50th	75th
Number of Attempts	McGrath	1.00	1.00	2.00
	HugeMed	1.00	1.00	2.00
Independent Samples *t*-Test				
			Statistic	*p*
Number of Attempts	Mann–Whitney U		181	0.788
Note. Hₐ μ _McGrath_ ≠ μ _HugeMed_				
Frequencies of Number of Attempts				
Number of Attempts	Group	Counts	% of Total	Cumulative %
1	McGrath	10	25.6%	25.6%
	HugeMed	11	28.2%	53.8%
2	McGrath	7	17.9%	71.8%
	HugeMed	8	20.5%	92.3%
3	McGrath	2	5.1%	97.4%
	HugeMed	1	2.6%	100.0%

**Table 3:** The comparison of a successful first tracheal intubation attempt.

Contingency Tables				
		Successful First Attempt		
Group		Yes	No	Total
McGrath	Observed	10	9	19
	% within row	52.6%	47.4%	100.0%
HugeMed	Observed	11	9	20
	% within row	55.0%	45.0%	100.0%
Total	Observed	21	18	39
	% within row	53.8%	46.2%	100.0%
χ^2^ Tests				
		Value	df	*p*
χ^2^		0.0220	1	0.882
N		39		

**Table 4:** Comparison of Cormack–Lehane scores between and within groups.

	Group		Total	Test Syt.	*p* *
	HugeMed	McGrath			
CML Score Indirect Laryngoscopy					
1	10 (50.00)	11 (57.89)	21 (53.85)	2.023	0.0034
2A	8 (40.00)	8 (42.11)	16 (41.03)		
2B	2 (10.00)	0 (0.00)	2 (5.13)		
CML Score Direct Laryngoscopy					
1	1 (5.00)	2 (10.53)	3 (7.69)	3.605	0.462
2A	4 (20.00)	6 (31.58)	10 (25.64)		
2B	6 (30.00)	7 (36.84)	13 (33.33)		
3	7 (35.00)	4 (21.05)	11 (28.21)		
4	2 (10.00)	0 (0.00)	2 (5.13)		
Test syt.	−3.862	−3.539			
*p*	<0.001	<0.001			
Pogo IL (percentage)	88.75 ± 4.44	92.63 ± 6.09		151.0	0.002
Pogo DL (percentage)	80.75 ± 6.74	78.42 ± 12.48		178.5	0.749
Test syt.	−3.967	−3.654			
*p* **	<0.001	<0.001			

* Chi-square test, frequency, ** Wilcoxon test. CML: Cormack Lehane, IL: Indirect Laryngoscopy DI: Direct Laryngoscopy.

**Table 5:** Comparison of inter and intragroup hemodynamic, SpO_2_ and etCO_2_ variables.

	HugeMed		McGrath		Test Syt.	*p*
	Mean ± Sd	Median (Min–Max)	Mean ± SS	Median (Min–Maks)		
Pre-Induction Systolic Arterial Pressure (mmHg)	92.60 ± 10.42	90.00 (70.00–110.00)	101.32 ± 15.93	100.00 (80.00–139.00)	127.000	0.079 *
Post Induction Systolic Arterial Pressure (mmHg)	95.30 ± 9.98	95.50 (80.00–123.00)	94.89 ± 22.30	90.00 (66.00–154.00)	148.500	0.247 *
Post Intubation Systolic Arterial Pressure mmHg)	91.00 ± 11.34	90.00 (73.00–120.00)	98.42 ± 16.31	100.00 (65.00–135.00)	129.000	0.089 *
Test syt.	0.711		1.425			
*p* ***	0.701		0.491			
Pre-Induction Diastolic Arterial Pressure (mmHg)	49.55 ± 13.50	50.00 (30.00–78.00)	58.16 ± 14.61	60.00 (35.00–84.00)	124.500	0.065 *
Post Induction Diastolic Arterial Pressure (mmHg)	53.20 ± 10.87	54.00 (34.00–75.00)	51.95 ± 16.19	47.00 (30.00–90.00)	156.500	0.351 *
Post Intubation Diastolic Arterial Pressure (mmHg)	50.30 ± 14.42	47.50 (30.00–90.00)	54.63 ± 11.88	56.00 (28.00–75.00)	138.000	0.149 *
Test syt.	2.135		0.592			
*p* ***	0.344		0.744			
Pre-Induction Mean Arterial Pressure (mmHg)	69.85 ± 10.79	72.00 (50.00–91.00)	74.42 ± 15.39	72.00 (55.00–106.00)	169.000	0.569 *
Post Induction Mean Arterial Pressure (mmHg)	70.95 ± 11.86	72.00 (44.00–98.00)	74.58 ± 18.96	71.00 (42.00–126.00)	180.000	0.792 *
Post Intubation Mean Arterial Pressure (mmHg)	69.55 ± 15.43	63.50 (45.00–113.00)	72.95 ± 14.83	75.00 (40.00–98.00)	150.500	0.270 *
Test syt.	1.600		1.368			
*p* ***	0.449		0504			
Pre-Induction Heart Rate (beat/min)	139.70 ± 14.68	141.00 (110.00–167.00)	136.63 ± 22.51	134.00 (103.00–178.00)	0.507	0.615 **
Post Induction Heart Rate (beat/min)	128.65 ± 12.71	128.00 (100.00–145.00)	133.11 ± 15.99	134.00 (110.00–160.00)	−0.966	0.340 **
Post Entubation Heart Rate (beat/min)	131.10 ± 15.17	130.00 (100.00–156.00)	132.74 ± 16.14	135.00 (87.00–162.00)	−0.326	0.746 **
Test ist.	3.348		0.400			
*p* ****	0.051		0.674			
Pre-Induction Oxygen Saturation (SpO_2_)	97.85 ± 1.31	98.00 (95.00–100.00)a	98.32 ± 1.46	99.00 (94.00–100.00)	145.000	0.214 *
Post Induction Oxygen Saturation (SpO_2_)	98.75 ± 0.97	99.00 (96.00–100.00)ab	97.68 ± 3.97	99.00 (85.00–100.00)	177.000	0.728 *
Post Entubation Oxygen Saturation (SpO_2_)	98.75 ± 1.25	99.00 (95.00–100.00)b	99.16 ± 2.12	99.00 (96.00–106.00)	171.500	0.607 *
Test syt.	8.600		1.733			
*p* ***	0.014		0.420			
Pre-Induction end tidal carbon dioxide (mmHg)	25.15 ± 4.04	26.00 (16.00–31.00)	24.42 ± 5.32	24.00 (12,00–36.00)	0.484	0.632 **
Post-Induction end tidal carbon dioxide (mmHg)	28.15 ± 3.18	28.00 (20.00–34.00)	31.26 ± 7.40	32.00 (20.00–45.00)	−1.691	0.104 **
Post Entubation end tidal carbon dioxide (mmHg)	32.10 ± 4.81	32.00 (25.00–41.00)	35.74 ± 5.77	34.00 (27.00–46.00)	−2.142	0.039 **
Test syt.	12.826		15.316			
*p* ****	<0.001		<0.001			

* Mann–Whitney U test, ** independent two sample *t* test, *** Friedman test, **** repeated analysis of variance.

**Table 6:** Examining the relationship between the number of attempts within groups and the parameters.

	HugeMed		McGrath	
	Number of Attempts		Number of Attempts	
	r	*p*	r	*p*
Post Intubation Systolic Arterial Blood Pressure (mmHg)	0.221	0.349	0.088	0.720
Post Intubation Diastolic Arterial Blood Pressure (mmHg)	0.304	0.192	−0.004	0.987
Post Intubation Mean Arterial Blood Pressure (mmHg)	0.104	0.663	−0.165	0.499
Post Intubation Heart Rate (beat/min)	0.311	0.182	−0.348	0.144
Post Intubation Oxygen Saturation (SpO_2_)	−0.144	0.544	−0.130	0.596
Post Intubation End Tidal Carbon Dioxide (mmHg)	−0.280	0.233	0.179	0.463

r: Spearman’s rho corelation.

**Table 7:** Correlation analysis.

Correlations			
	Age (Years/Months)	POGO VL(%)	POGO DL(%)
Age (Years/Months)	1	0.228	−0.358
		0.347	0.132
	19	19	19
	1	0.340	0.103
		0.132	0.658
	21	21	21

## Data Availability

Data can be shared upon request.
